# The influence of difficult child temperament and parenting stress on parental problematic smartphone use in early childhood: a moderated mediation analysis

**DOI:** 10.3389/fpsyg.2025.1517222

**Published:** 2025-05-27

**Authors:** Larissa Schneebeli, Katrin Braune-Krickau, Anouk Joliat, Agnes von Wyl

**Affiliations:** ^1^School of Applied Psychology, ZHAW Zurich University of Applied Sciences, Zürich, Switzerland; ^2^School of Health Sciences, ZHAW Zurich University of Applied Sciences, Winterthur, Switzerland

**Keywords:** parental smartphone use, problematic smartphone use, difficult child temperament, parenting stress, early childhood

## Abstract

Smartphone use has become an integral part of daily life and is also common among parents of young children. As parental problematic smartphone use can impact parents’ and children’s well-being, it is important to examine the determinants of this issue. This study examined the association between difficult temperament in toddlers, parenting stress, and parental problematic smartphone use. Results of a moderated mediation analysis utilizing data from 261 mothers and fathers of 14-month-old children showed that parenting stress fully mediated the association between difficult child temperament and parental problematic smartphone use. Parental gender did not moderate the association between parenting stress and problematic smartphone use. Parents who rated their child’s temperament as more difficult reported more parenting stress, which in turn led to higher levels of problematic smartphone use. Parenting stress is thus an important determinant of parental problematic smartphone use. Further research on stress management and parental smartphone use is needed to develop targeted interventions that promote the well-being of parents and children. Parental smartphone use should be considered holistically by taking various influencing factors and their interplay into account rather than examining them in isolation.

## 1 Introduction

Smartphones have become omnipresent in everyday life (Schnauber-Stockmann and Mangold, 2020). Smartphone use may have a positive impact on aspects such as well-being for some individuals (Lavoie and Zheng, 2023), but it can also have a negative impact on social interactions. Interindividual studies have found negative associations between smartphone use and the quality of couple relationships (e.g., McDaniel and Coyne, 2016), parent–child interactions (for an overview, see Braune-Krickau et al., 2021), and children’s behavior problems (Kim et al., 2022; McDaniel and Radesky, 2018a; McDaniel and Radesky, 2018b; Barr et al., 2020). Additionally, smartphone use can become problematic and have a negative impact on a person’s well-being. Studies with an intraindividual focus have found a negative association between smartphone use and mental health (for an overview, see Yang et al., 2019).

Different aspects of smartphone use may have negative effects on psychological well-being and interaction quality, such as smartphone screen time (Anderl et al., 2024). However, smartphone screen time alone is not a reliable indicator of well-being (Barr et al., 2020; Buda et al., 2023; David et al., 2017). Problematic smartphone use, but not smartphone screen time, has been found to correlate with mental health problems like depression and states of anxiety (Rozgonjuk et al., 2018). However, there is as yet no standardized definition for problematic smartphone use (Sánchez-Fernández and Borda-Mas, 2023). It is often referred to as “smartphone addiction” in reference to behavioral addictions (Panova and Carbonell, 2018). It is important to note that the term implies a diagnosis that does not actually exist (American Psychiatric Association [APA], 2022). Kwon et al. (2013) have described problematic smartphone use as excessive (screen time), compulsive, and uncontrolled use. According to a meta-analysis examining problematic smartphone use in persons aged 15 to 35 in several countries using the same instrument (smartphone addiction scale), there has been a rise in problematic smartphone use globally (Olson et al., 2022).

But what contributes to problematic smartphone use? The determinants can be diverse. Studies have reported associations between problematic smartphone use and psychopathological symptoms, including anxiety (Alavi et al., 2020; Demirci et al., 2015; Jenaro et al., 2007; Kim et al., 2019; Kwak and Kim, 2023; Lee et al., 2016; Rochat et al., 2025) and depression (Alavi et al., 2020; Augner and Hacker, 2012; Demirci et al., 2015; Harwood et al., 2014; Kalaitzaki et al., 2024; Kim et al., 2019; Thomée et al., 2011; Yuan et al., 2021). Additionally, associations have been found with sleep problems (Alzhrani et al., 2023; Demirci et al., 2015; Sohn et al., 2021; Thomée et al., 2011), low self-control (Kwak and Kim, 2023), loneliness (Laurence et al., 2020), and having underage children (Rochat et al., 2025).

During the child’s toddler years, additional factors could contribute to problematic smartphone use in their parents. Parenthood brings with it numerous new responsibilities and challenges (Benz and Scholtes, 2015), and stressful situations are often a part of daily life with young children (Deater-Deckard, 2004). In the first three years of life, development proceeds at the fastest pace, and the most profound changes occur (Pauen et al., 2012). Parents must adapt flexibly and quickly to the new phases of development. In their second year of life, children make strides in autonomy, motor development, and communication (Pauen and Roos, 2020); these steps in development require a great deal of adaptation on the part of the parents. This can increase parenting stress. Parenting stress refers to a distinct form of stress linked to the experience of being a parent (Abidin, 1992; Diener and Swedin, 2020) and may arise when parents perceive subjectively that they do not have adequate resources to cope with the demands of parenting (Deater-Deckard, 1998). A German study on early childhood (0–3 years) has found that parenting stress follows a U-shaped curve (Fullerton et al., 2019): In the first months of a child’s life, parents experience increased stress, which then decreases when the child is about 6 months old. Stress then rises again from the second year of life onwards (Fullerton et al., 2019). Parenting stress is thus strongly associated with the child’s age and development. However, stress is also a subjective experience (Diener and Swedin, 2020). There may also be differences between mothers and fathers in terms of parental stress. Some studies have shown that mothers experience higher levels of parenting stress compared to fathers (Delvecchio et al., 2015; Hildingsson and Thomas, 2014; Liu and Wang, 2015; Rayce et al., 2020; Skreden et al., 2012; Tedgard et al., 2020). In contrast, other studies have found that fathers report greater parenting stress (Vismara et al., 2021) or that there are no significant differences between mothers and fathers (Azzi et al., 2023). Parenting stress could lead parents to flee into the world of the smartphone. Indeed, parents may turn to the smartphone as a way of coping with stress (Radesky et al., 2016; Wolfers, 2021). There is also a positive association between experiencing stress and problematic smartphone use (Chiu, 2014; Gökçearslan et al., 2018; McDaniel and Radesky, 2018b; Tu et al., 2023).

Another factor that might affect parents’ smartphone use or contribute to their stress level is difficult child temperament. Temperament describes individual differences in the response to internal and external stimuli as well as in self-regulation strategies (Rothbart and Bates, 2006). Temperament-related traits are evident early in behavior and are reflected in activity level, mood, attention span, and self-regulation (Rothbart, 2011). Temperament emerges from a complex interplay of genetic, biological, and environmental factors (Rothbart and Bates, 2006). The neurobiological developmental approach (Rothbart, 1981; Rothbart and Bates, 2006) differentiates between three temperament dimensions: Surgency/Extraversion, Orienting/Regulation, and Negative Affectivity (Gartstein and Rothbart, 2003). A temperament perceived as difficult could be characterized by high negative affectivity and low regulatory competence in children. Research indicates that these traits are associated with increased parental stress (Oddi et al., 2013; Olafsen et al., 2008; Pesonen et al., 2008). In the child psychiatric approach by Thomas and Chess (1977) there are three child temperament types based on nine dimensions of infant temperament: the easy child, the difficult child, and the slow-to-warm-up child. Children with a difficult temperament are usually negative in mood, have trouble adapting to routine and new situations, have irregular patterns of eating, sleeping, etc., and low self-regulation (Thomas et al., 1982). The temperament types not only differ in the child’s behavioral tendencies but can also elicit different behavioral reactions in parents (Bradley and Corwyn, 2019; Kivijärci et al., 2005). Indeed, child temperament and parenting mutually influence each other (Kiff et al., 2011; Micalizzi et al., 2015).

Therefore, a difficult child temperament can make parenting especially challenging and demanding of even more resources, as in parenthood there is in any case a clash between children’s and parents’ needs (Nystrom and Ohrling, 2004). In fact, a difficult child temperament has been associated with lower parental well-being, including higher levels of anxiety (Britton, 2011), depression (Britton, 2011; Solmeyer and Feinberg, 2011), lower levels of self-reported parental efficacy (Solmeyer and Feinberg, 2011; Takács et al., 2019), and increased parenting stress (Solmeyer and Feinberg, 2011; McBride et al., 2004; Östberg and Hagekull, 2000). Therefore, parents could turn to the smartphone to meet their own needs for autonomy or social connections (Ryan and Deci, 2000). They use their smartphones to take some time for themselves in everyday life (Radesky et al., 2016; Wolfers, 2021) or seek social support or connect with others socially (Coyne et al., 2022; Dworkin et al., 2013; McDaniel et al., 2012) through social networks and communication platforms. Parents also use smartphones as a source of information (Coyne et al., 2022; Dworkin et al., 2013; Radesky et al., 2016) on parenting or child development.

In summary, it can be assumed that both difficult child temperament and parental stress may be positively associated with parental problematic smartphone use, and parental stress may potentially play a mediating role. Given gender-specific differences in the perception of parental stress and problematic smartphone use, parent gender may also be a moderating factor.

**Figure 1:**
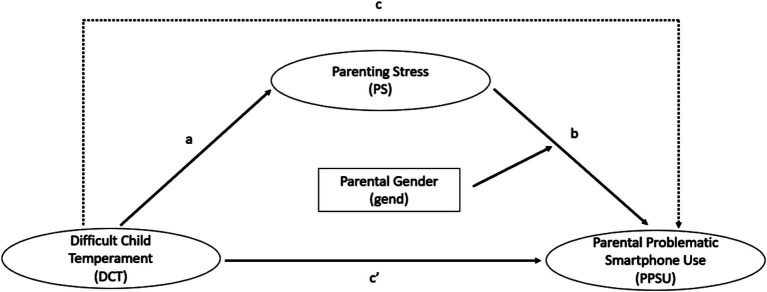
Conceptual model with direct (solid arrows) and indirect (dashed arrows) associations. As our study employs a cross-sectional design, we refer to associations (direct, indirect, and total association) rather than effects (direct, indirect, and total effect).

However, research on this topic remains limited, particularly in early childhood—a crucial developmental phase. The first three years of life are formative for a child’s cognitive, emotional, and social development (Pauen and Roos, 2020), and young children are highly dependent on their parents’ physical and mental presence (Biringen and Easterbrooks, 2012). Parents play a crucial role in supporting their children’s developmental milestones, such as emotional regulation (Holodynski, 2006; Tronick, 1989). Therefore, understanding how parenting stress and difficult child temperament are associated with problematic smartphone use in parents of young children is essential to prevent potential negative effects on parent well-being and on parent–child interactions. This study aimed to explore these determinants within this context. In parents with 14-month-old children, we examined whether there is an association between difficult child temperament and parental problematic smartphone use and whether this association is mediated by parenting stress. Additionally, we aimed to examine the role of parent gender. Figure 1 shows our conceptual model. Based on the research evidence reviewed above, we formulated the following hypotheses:

*Hypothesis 1*: The association between difficult child temperament and parental problematic smartphone use is mediated by parenting stress.

*Hypothesis 2*: The association between parenting stress and parental problematic smartphone use is moderated by parent gender.

## 2 Materials and methods

### 2.1 Participants and procedures

This study utilized data from the Smart Toddlers study, a study conducted in Switzerland on parental smartphone use, parent–child interactions, and child development in the toddler years (aged 14–36 months). Smart Toddlers is the follow-up study to the Smart Start study (Wade-Bohleber et al., 2024); some parents in that study participated in the Smart Toddlers study as well. Other parents were recruited through health institutes, child care facilities, social media channels, and a magazine advertisement in the German-speaking part of Switzerland. Inclusion criteria were: participation with a first-born child, resident of Switzerland, no chronic diseases, use of digital media, and sufficient German or English language skills to fill out the questionnaires, which were available in German and English. For this study we used Smart Toddlers study data from time point T1 (14 months postnatal), in which 261 parents had participated. Of these 261 parents, 110 couples and 23 mothers with a first-born child had participated. Of the 119 couples, one was a same-sex couple. Together with this couple, one mother was defined as a co-mother. One couple participated with her twins, whereby we used the data only from the older twin in the study. Table 1 shows characteristics of the sample.

**Table 1:** Demographics of parents and children.

Parents (*n* = 261)	Mothers (*n* = 142)	Fathers/Co-mothers (*n* = 119)
Mean age in years (SD)	*n* = 141	*n* = 114
	34.03 (3.65)	37.05 (5.87)
Swiss nationality (in %)*	*n* = 142	*n* = 119
	82.4	73.9
Highest education degree (in %)	*n* = 142	*n* = 119
No completed education	-	-
Compulsory education^1^	-	-
Vocational education: Apprenticeship^2^	10.6	19.3
Maturity certificate^2^	2.8	4.2
Higher vocational education^3^	17.6	11.8
University degree^3^	69	64.7
Employment rate (in %)	*n* = 141	*n* = 115
100%	7.1	49.6
≥ 80%	15.6	38.3
≥ 50%	44.0	7.0
< 50%	24.8	3.5
Variable	2.1	
Unemployed	6.4	1.5
Financial pressure (worry) in %	*n* = 141	*n* = 114
1 = no, not at all	34.0	33.3
2	32.8	41.2
3	20.6	19.3
4	11.3	5.3
5 = yes, very much so	1.4	0.9
Children (*n* = 142)		
Mean age in months (SD)	14.46 (0.62)	
Sex (female in %)	49.4	

*Including dual nationality. ^1^Lower secondary level of education in Switzerland; ^2^upper secondary level of education in Switzerland; ^3^tertiary level of education in Switzerland (ISCED 97 levels 5 A, 5 B, and 6).

Prior to the start of the study, all participants were informed about the study aim and procedures and signed a participation consent form. Study participants received no financial compensation for their participation.

### 2.2 Instruments

When their child was 14 months old, parents received a link by email to the online questionnaire.

#### 2.2.1 Difficult child temperament

To assess difficult infant temperament, or rather parent perceptions of their infants’ difficultness, we used the Infant Characteristics Questionnaire (ICQ; Bates et al., 1979). For this study we used the nine items in the subscale Fussy/Difficult from the ICQ version for infants aged about 13 months. The nine items evaluate the infant’s crying, ability to be soothed, mood, and challenges encountered in typical day-to-day situations. For example, three of the nine items were: (1) How much does your baby cry and fuss in general?, (2) How changeable is your baby’s mood?, and (3) How persistent is your baby in trying to get your attention when you are busy?. The nine items were measured on a 7-point Likert scale, with a higher score corresponding to a more difficult infant temperament. Bates et al. (1979) reported an internal consistency of the subscale Fussy/Difficult of *α* = 0.79. Cronbach’s alpha in the current study was *α* = 0.82.

#### 2.2.2 Parenting stress

Parenting stress was assessed using the parent domain of the German version (*Eltern-Belastungs-Inventar*; EBI) of the Parenting Stress Index (PSI; Abidin, 1995), which includes the following seven subscales with a total of 28 items (Tröster, 2011): (1) attachment, (2) isolation, (3) competence, (4) depression, (5) health, (6) role restriction, and (7) spouse/parenting partner relationship. Questions including “I sometimes have the feeling that I cannot handle some things very well” or “I sometimes feel trapped by my responsibilities as a parent” are scored on a 5-point Likert scale with anchors 1 = strongly disagree and 5 = strongly agree. Higher scores indicate a higher level of parenting stress. Tröster (2011) reported a reliability coefficient of *α* = 0.93 for the parent domain. In this study, the internal consistency was *α* = 0.90.

#### 2.2.3 Parental problematic smartphone use

We employed seven items to assess parental problematic smartphone use, which we adapted from Barnes et al.’s (2019), Addiction to the Device scale by specifically focusing on the smartphone in each item. The translation into German was done by a bilingual English and German speaker (native speaker in both languages) and subsequently reviewed by someone proficient in both German and English. The items focused on identifying addictive behaviors and emotions related to smartphone usage, specifically: (1) I sometimes neglect important things because of my interest in my smartphone, (2) My social life has sometimes suffered because of me interacting with my smartphone, (3) Using my smartphone sometimes interfered with other activities, (4) When I am not using my smartphone, I often feel agitated, (5) I have made unsuccessful attempts to reduce the time I use my smartphone, (6) I feel lost without my smartphone, and (7) I tend to get easily distracted by my smartphone. Participants responded to the items on a 7-point Likert scale, with anchors 1 = strongly disagree and 7 = strongly agree. Barnes et al. (2019) reported an internal consistency of *α* = 0.84. In this study, Cronbach’s alpha was *α* = 0.83.

### 2.3 Data analysis

Statistical analyses were conducted using SPSS Version 29.0.1 and RStudio Version 2023.09.1. First, we calculated the descriptive statistics of the variables of interest and then computed Pearson correlation coefficients among the scale mean scores. To reduce the complexity of the further analysis, the number of items for the three constructs parental problematic smartphone use, difficult child temperament, and parenting stress was reduced through item parceling. Item parceling has been used more and more frequently in recent years (see Bandalos, 2002; Hapsari and Widhiarso, 2023; Little et al., 2002; Hau and Marsh, 2004; Marsh et al., 1998). Initially, confirmatory factor analyses (CFA) were conducted to verify the theoretically assumed measurement models for the constructs of parental problematic smartphone use, difficult child temperament, and parenting stress prior to item parceling. For the unidimensional constructs parental problematic smartphone use and difficult child temperament, a CFA with a single-factor model was conducted for each. For the multidimensional construct parenting stress, a seven-factor model was specified, with items assigned to seven latent dimensions (PS1 to PS7) according to their theoretical allocation. The results of the CFA (see Appendix Table A1) indicate that an optimal model fit was not achieved for all constructs. Specifically, the root mean square error of approximation (RMSEA) for the constructs parental problematic smartphone use and difficult child temperament exceeds the recommended thresholds (Hu and Bentler, 1999). However, according to Kenny et al. (2014), this can occur in models with a low number of degrees of freedom. All items show significant and predominantly acceptable factor loadings (mostly > 0.50), indicating satisfactory conceptual coherence of the respective constructs. Given the planned use of item parcels for the subsequent analyses, all items, including those with low factor loadings, were retained due to their theoretical relevance. Based on this decision, and adapted from Little et al. (2002), the items for the unidimensional constructs parental problematic smartphone use and difficult child temperament were divided into three parcels using the balancing approach of item scale correlation. The items were divided such that the item with the highest and the item with the lowest correlation formed a parcel, the second highest with the second lowest a parcel, and the remaining three items another parcel. For the construct difficult child temperament, the item with the highest, the lowest, and the third-highest item-scale correlation formed a parcel, and so on. For the multidimensional construct parenting stress, we used domain representative parceling: Here four parcels were created with one item each from each dimension (Little et al., 2002). Cronbach’s alpha of the parcels for parental problematic smartphone use, difficult child temperament, and parenting stress was *α* = 0.83, *α* = 0.82, and *α* = 0.92, respectively. To ensure internal consistency, the parcel discriminations were considered. These ranged from 0.65 to 0.72 for difficult child temperament, from 0.78 to 0.84 for parenting stress, and from 0.69 to 0.70 for parental problematic smartphone use, indicating good scale reliability (Bortz and Döring, 2006). We then conducted a moderation mediation analysis using latent variables, based on structural equation modeling (SEM) using lavaan (Rosseel, 2012) with the maximum likelihood robust (MLR) estimator, which provides robust standard errors. To further account for the nested data structure, we applied cluster-robust standard errors (CRSE; Cameron and Miller, 2015), which correct for within-cluster dependencies in standard error estimation.

The moderated mediation analysis was conducted with parental problematic smartphone use as the outcome variable, difficult child temperament as the predictor variable, and parenting stress as the mediator. Parental gender was modeled as the moderator of the association between parenting stress and parental problematic smartphone use. The latent interaction variable was modeled using double mean centering (Lin et al., 2010). As a covariate we had wanted to include parental education as a binary variable (0 = no university degree, 1 = university degree) to measure family socioeconomic status (SES), as research has shown it to be the most consistent and widespread component of SES (Sirin, 2005). However, as parental education did not significantly correlate with any other variable (see Table 2), we did not include it in the model. As model fit indices, we report the relationship between the chi-square (*χ*^2^) and degrees of freedom (*df*), the comparative fit index (CFI), the root mean square error of approximation (RMSEA), and the standardized root mean square residual (SRMR). CFI values over 0.95, RMSEA values below 0.06, and SRMR values less than 0.08 indicated a good model fit (Hu and Bentler, 1999).

**Table 2:** Bivariate correlations among manifest study variables.

Variable	1	2	3
(1) PPSU			
(2) DCT	0.16*		
(3) PS	0.36***	0.37***	
(4) edu	−0.01	0.10	−0.01

PPSU, parental problematic smartphone use; DCT, difficult child temperament; PS, parenting stress; edu, education. ^∗^*p* < 0.05; ^∗∗^*p* < 0.01; ^∗∗∗^*p* < 0.001.

## 3 Results

### 3.1 Descriptive statistics

Table 2 presents bivariate correlations among the variables in this study, Table 3 the descriptive statistics for the variables.

**Table 3:** Descriptive statistics of the variables of interest.

Variable	Mothers					Fathers / Co-mothers					*p*
	*n*	*M*	*SD*	Min	Max	*n*	*M*	*SD*	Min	Max	
PPSU	140	3.04	1.09	1.00	5.57	113	3.15	1.08	1.00	5.86	0.392
DCT	140	3.44	0.84	1.56	6.22	113	3.43	0.83	1.44	5.89	0.896
PS	139	2.75	0.60	1.18	4.39	112	2.67	0.61	1.29	4.21	0.260

The mean values are calculated from the manifest variables. PPSU, parental problematic smartphone use; DCT, difficult child temperament; PS, parenting stress. The *p*-values represent the results of independent samples *t*-tests comparing mothers and fathers/co-mothers across the three variables.

No significant mean differences were found between mothers and fathers for the variables problematic smartphone use, difficult child temperament, and parenting stress. For parenting stress, 46% of mothers and 37.5% of fathers had a high level according to the test manual (*t*-value ≥ 60), which can lead to difficulties in coping with parenting tasks (Tröster, 2011).

### 3.2 Moderated mediation analysis

**Figure 2:**
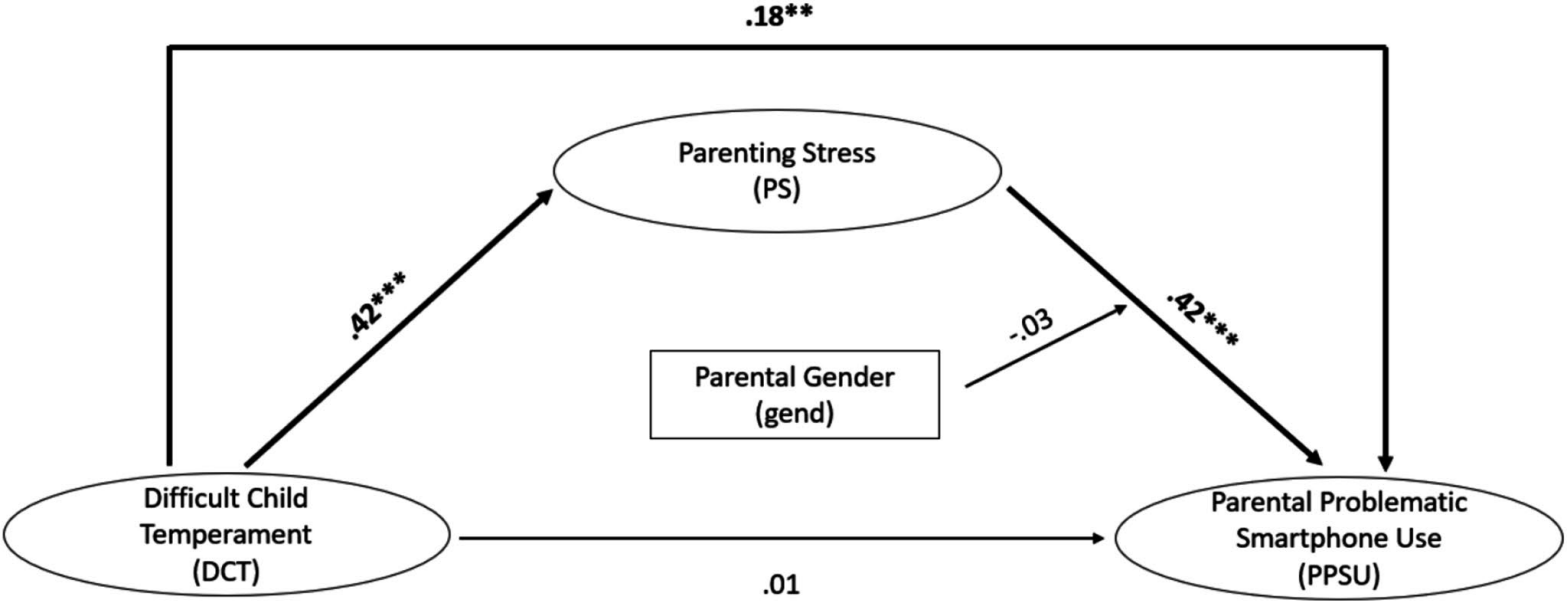
Moderated mediation. *p < 0.05, **p < 0.01, ***p < 0.001. *n* = 253; number of clusters = 142, χ^2^ = 114.66; *df* = 85; CFI = 0.99; RMSEA = 0.037; SRMR = 0.035.

The model was found to fit the data well. For information on the model fit indices (scaled) and the standardized results, see Figure 2 and Appendix Table A2.

Difficult child temperament was significantly positively associated with parenting stress (*ß* = 0.42, *p* < 0.001). The more difficult the child’s temperament was rated, the higher the reported parenting stress was. In turn, parenting stress was significantly positively associated with parental problematic smartphone use (*ß* = 0.42, *p* < 0.001). The greater the extent to which parents reported experiencing parenting stress, the higher the level of parental problematic smartphone use was. Parent gender did not moderate the association between parenting stress and parental problematic smartphone use (*ß* = −0.03, *p* = 0.607). There was no direct association between difficult child temperament and parental problematic smartphone use (*ß* = 0.01, *p* = 0.956). The indirect association between difficult child temperament and parental problematic smartphone use, mediated by parenting stress, was statistically significant (*β* = 0.18, *p* = 0.001). This means that parenting stress acted as a mediator. The total association between difficult child temperament and parental problematic smartphone use was significant (*β* = 0.18, *p* = 0.023) as well. When examining the indirect association in a model differentiated by the gender subgroup [*χ^2^*(64) = 79.07; CFI = 0.99; RMSEA = 0.043; SRMR = 0.040], we observed a significant indirect association for both mothers (*β* = 0.18, *p* = 0.016) and fathers (*β* = 0.17, *p* = 0.039). However, the difference between genders was not significant (*p* = 0.892).

## 4 Discussion

This study examined two determinants of problematic smartphone use in parents of toddlers: difficult child temperament and parenting stress. There was a significant association between difficult child temperament and parental problematic smartphone use, which was fully mediated by parenting stress, thereby confirming hypothesis 1. Specifically, parents of 14-month-old children who perceived their child’s temperament as more difficult reported higher levels of parenting stress, which in turn was linked to increased problematic smartphone use. Further, parent gender did not moderate the association between parenting stress and parental problematic smartphone use. Consequently, hypothesis 2 was not supported.

### 4.1 Difficult child temperament and parenting stress

The more difficult a child’s temperament was rated by their parents, the more parenting stress the parents reported. Consistent with prior research demonstrating the association between difficult child temperament and parenting stress (e.g., Chang et al., 2004; McBride et al., 2004; Molfese et al., 2010; Östberg and Hagekull, 2000; Solmeyer and Feinberg, 2011), parents reported increased levels of parenting stress when they perceived their child as irritable and difficult to soothe. Shin et al. (2021) demonstrated that higher negative affectivity and lower effortful control in toddlers were associated with increased parenting stress. Parenting stress can arise among other things due to feeling overwhelmed in parenting, having a feeling of low self-efficacy in the parent role, and experiencing personal restrictions imposed by parenthood (Deater-Deckard, 2004; Diener and Swedin, 2020). The behavior of a 14-month-old child with a temperament perceived as difficult could intensify these stressors. For instance, Porter and Hsu (2003) found that mothers who perceived their infants as having a difficult temperament reported lower maternal efficacy.

In addition, parenting stress can restrict parental mentalization. A central assumption of the mentalization approach is that the ability to mentalize decreases when stress increases (Luyten and Fonagy, 2015). Parental mentalization refers to the capacity of parents to think about their own and their child’s behaviors in the context of mental states (needs, desires, feelings, etc.) (Fonagy et al., 2015). If parents are not able to mentalize, they do not see difficult behaviors as caused by their child’s temperament; instead, they may feel that the behavior is directed against themselves, which in turn can lead to feeling low self-efficacy as a parent and a higher stress level. This could have a negative effect on the child’s behavior and result in a vicious circle.

The goodness of fit model by Thomas and Chess (1977) could also be relevant. The model, which describes the fit between parents’ behavior and the child’s temperament, describes how parents manage and respond to their child’s temperament. High goodness of fit means that there is a strong match between the child’s temperament and parents’ expectations, requirements, and behaviors, so that parents can deal better with the challenges of a child’s difficult temperament. This can reduce the stress that parents experience. Low goodness of fit means that the parents find the child’s needs and behaviors very difficult, which can lead to a higher level of stress. The goodness of fit may therefore also play a role in the experience of parenting stress.

### 4.2 Parenting stress and parental problematic smartphone use, and the role of parent gender

Parents of 14-month-old children who reported a higher level of parenting stress also scored high on problematic smartphone use. This is consistent with Chiu (2014), Gökçearslan et al. (2018), and Tu et al. (2023), who also found positive associations between stress and problematic smartphone use independent of parenting. A possible explanation of the association could be that parents with higher stress levels use their smartphones as a coping strategy, for example for social support (Radesky et al., 2016), to reduce feelings of isolation (Coyne et al., 2022), or to take some time for themselves (Wolfers, 2021). Coping is defined as the cognitive and behavioral efforts a person employs to manage internal and external stressful situations (Lazarus and Folkman, 1984). Lazarus and Folkman’s (1984) transactional model of stress and coping focuses on three coping strategies: perception-based coping strategies, problem-based coping strategies, and emotion-based coping strategies. In coping with parenting stress by using a smartphone, the question arises as to what people are doing on their smartphones. Two qualitative studies (Torres et al., 2021; Wolfers, 2021) found that parents primarily use their smartphones for information, self-distraction, and social support. Acquiring information, such as tips on parenting when children’s behavior is challenging, can be assigned to problem-oriented coping. Self-distraction and social support are more emotion-focused coping strategies. However, smartphone-based coping strategies have been found to be less effective in reducing stress than other strategies (Wolfers et al., 2023).

Parent gender did not moderate the association between parenting stress and problematic smartphone use. This means that the effect of parenting stress on problematic smartphone use does not differ between mothers and fathers. Both mothers and fathers may turn to their smartphones as a coping mechanism when experiencing increased parenting stress. Due to social change, fathers – for example in Germany [Bundesministerium für Familie, Senioren, Frauen und Jugend (BMFSFJ), 2021] – are increasingly involved in child care, and in our sample, almost 50% of fathers worked part-time. This means that fathers as well as mothers have to develop coping strategies for parenting stress. We can also assume that the couple relationship influences smartphone behavior and could lead to similar coping strategies in mothers and fathers. McDaniel and Radesky (2018b) found that there is a positive correlation between problematic digital technology use in mothers and in fathers.

Our finding that parent gender did not have a moderating effect only partially agrees with Tu et al.’s (2023) results: They found that among high school students, gender had no moderating effect on stress and information acquisition addiction but did have a moderating effect on stress and social media addiction (significantly higher in females) and game addiction (significantly higher in males). The different operationalizations of problematic smartphone use as well as the age of the students could play a role here.

### 4.3 Difficult child temperament and parental problematic smartphone use, and parenting stress as a mediator

The total association indicated that difficult child temperament has a significant overall impact on parental problematic smartphone use. However, this significant association was fully mediated by parenting stress. These findings are consistent with McDaniel and Radesky (2018a), who also identified parenting stress as a mediator between children’s behavioral problems and parental smartphone use. Parenting stress can arise from the daily challenge of handling the many tasks and responsibilities in child care as well as other obligations (Diener and Swedin, 2020). Parenting made more challenging due to difficult child temperament could result in parents being less able to satisfy their own needs for competence, autonomy, and relatedness, which in turn increases parenting stress. Parents possibly turn to their smartphone as an escape from this stress and to satisfy their needs at least in the short term, which in addition to other factors can lead to a higher level of problematic smartphone use. As previously mentioned, this study did not find a direct association between difficult child temperament and parental problematic smartphone use. This does not agree with the findings of the Wade-Bohleber et al. (2024) study, in which a partial sample of our study sample also took part. Wade-Bohleber et al. (2024) found a positive association between difficult child temperament and parental problematic smartphone use in the first months of a child’s life. They assumed that parents of a child with a difficult temperament experience less autonomy and social connectedness and that smartphone use has a compensatory function (Wade-Bohleber et al., 2024). According to Ryan and Deci’s (2000) self-determination theory, the need for competence, autonomy, and relatedness are basic human psychological needs. As the children in the Wade-Bohleber et al. (2024) study were considerably younger than the children in our study, this could indicate that over time, the parents had found other ways to satisfy those needs. It could also be that they found no other strategies and that their unsatisfied needs led to more parenting stress, for example feeling more restricted personally. That would explain our finding that parenting stress mediates the association between difficult child temperament and parental problematic smartphone use. In sum, the results show that parenting stress, which can vary depending on the child’s temperament, is a decisive determinant in problematic smartphone use of parents of toddlers.

### 4.4 Strengths and limitations

To our best knowledge, this is the first study to investigate whether parenting stress acts as a mediator between child temperament and parental problematic smartphone use and taking potential gender moderation into account. Paternal data were also considered, which are often neglected in studies on this topic. Another strength of this study is that the questionnaires were provided in both German and English, making it possible for immigrants to participate in the study and increasing the diversity in our sample. In summary, this study represents a unique contribution to our understanding of the determinants of parental problematic smartphone use in early childhood.

We must also mention some limitations of the study. One limitation is the high education level of the study sample: A high percentage of mothers (69%) and fathers (64.7%) had university degrees. However, for the year 2022, statistics collected by the Federal Statistical Office [Bundesamt für Statistik (BfS), 2023] indicate that only 35.5% of women and 34.3% of men aged 35–44 years had a university degree. A higher education level is therefore overrepresented in our sample. In addition, a tendency toward self-selection in the sample cannot be excluded. For example, the parents who decided to participate in the study may have had an interest in and curiosity about the topic of the study as well as a certain awareness regarding smartphone use.

Another limitation is that although cross-sectional studies can identify correlations between variables, they cannot clearly determine the direction of causality. For example, it cannot be excluded that the indirect association between difficult child temperament and parental problematic smartphone use may be bidirectional. This would mean that a higher level of problematic smartphone use leads to more parenting stress, which in turn leads to more negative behavior in the child, such as more negative affect. This behavior could in turn be perceived by parents as difficult child temperament.

In addition, parental problematic smartphone use, difficult child temperament, and parenting stress were measured by parents’ self-report. In general, self-report surveys have the disadvantage that the results may possibly be affected by participants giving socially desirable responses (Paulhus, 2017). We did not measure social desirability bias in this study.

Further, the results of the confirmatory factor analyses indicate that an optimal model fit was not achieved for some constructs. These suboptimal model fits could impair the validity of the results, as they suggest that the measurement models may not fully capture the underlying theoretical constructs. This could affect the reliability and accuracy of the measurements, thereby limiting the interpretability and generalizability of the findings.

### 4.5 Conclusion and implication for practice

This cross-sectional study found that parenting stress fully mediates the positive association between difficult child temperament and problematic smartphone use in parents of 14-month-old children. The findings highlight the importance of considering parenting stress as a central mechanism in understanding the link between child temperament and parents’ smartphone use behaviors.

The results underline that—in both research and practice—parental problematic smartphone use (and smartphone use in general) should not be viewed in isolation. Instead, it should be considered an integrated component of a comprehensive and complex system and analyzed in relation to parents’ broader context. This means that for a comprehensive understanding of parental smartphone use and the development of effective interventions, smartphone use should be examined in the context of life circumstances, stress factors, and stress coping strategies. Furthermore, the increasing digitalization of everyday life and the associated social pressure to always be available should not be overlooked.

These interventions should not primarily convey to parents that they should reduce their smartphone use but should focus instead on how they can manage their stress effectively. Approaches may include promoting mindfulness, facilitating peer support among parents, organizing parent groups, or providing targeted counseling services. Studies have shown, for example, that mothers were able to reduce their parenting stress through a Mindful Parenting Training (Fernandes et al., 2022). The overarching goal is to foster *good enough* parenting (Winnicott, 1953), thereby supporting both healthy child development and parents’ well-being.

### 4.6 Future directions

Future studies should employ a bidirectional longitudinal design to clarify the direction of causality. This would help determine whether parental smartphone use also influences children’s behavior in the context of temperament. As both fathers and mothers exhibit problematic smartphone use behaviors in connection with parenting stress, it is important to include both fathers and mothers in future research. Furthermore, targeted guidelines and counseling services could be developed to provide parents with strategies for managing challenging situations in early childhood and effective methods for coping with stress. Adequate stress management is particularly relevant in light of the fact that smartphone use as a coping strategy is less effective in reducing stress than other strategies (Wolfers et al., 2023). Finally, the concept of problematic smartphone use is widely used but lacks a consistent definition. Future research should aim to establish clear and standardized definitions to enhance comparability across studies.

## Data Availability

The raw data supporting the conclusions of this article will be made available by the authors, without undue reservation.
