# Supplementary material for: The influence of difficult child temperament and parenting stress on parental problematic smartphone use in early childhood: a moderated mediation analysis
Source: Front Psychol. 2025 May 27;16:1517222. doi: 10.3389/fpsyg.2025.1517222 (PMC12149099; doi:10.3389/fpsyg.2025.1517222)
Supplement: Supplementary file 1 [file Table_1.docx]

**Appendix A. Supplementary data**

**Table A.1**Model fit indices and standardized factor loadings from the confirmatory factor analyses.

| **Construct** | **Item** | ***χ² (df)*** | **CFI** | **RMSEA** | **SRMR** | **Factor Loadings** |
| --- | --- | --- | --- | --- | --- | --- |
| **PPSU** |  | 61.17 (14) | .909 | .117 | .060 |  |
|  | PPSU1 |  |  |  |  | .71 |
|  | PPSU2 |  |  |  |  | .53 |
|  | PPSU3 |  |  |  |  | .74 |
|  | PPSU4 |  |  |  |  | .62 |
|  | PPSU5 |  |  |  |  | .60 |
|  | PPSU6 |  |  |  |  | .48 |
|  | PPSU7 |  |  |  |  | .73 |
| **DCT** |  | 118.12 (27) | .854 | .117 | .076 |  |
|  | DCT1 |  |  |  |  | .19 |
|  | DCT2 |  |  |  |  | .53 |
|  | DCT3 |  |  |  |  | .76 |
|  | DCT4 |  |  |  |  | .65 |
|  | DCT5 |  |  |  |  | .66 |
|  | DCT6 |  |  |  |  | .56 |
|  | DCT7 |  |  |  |  | .52 |
|  | DCT8 |  |  |  |  | .50 |
|  | DCT9 |  |  |  |  | .73 |
| **PS** |  | 609.94 (329) | .853 | .059 | .072 |  |
| PS1 | PS11 |  |  |  |  | .66 |
|  | PS12 |  |  |  |  | .72 |
|  | PS13 |  |  |  |  | .69 |
|  | PS14 |  |  |  |  | .23 |
| PS2 | PS21 |  |  |  |  | .74 |
|  | PS22 |  |  |  |  | .59 |
|  | PS23 |  |  |  |  | .39 |
|  | PS24 |  |  |  |  | .39 |
| PS3 | PS31 |  |  |  |  | .68 |
|  | PS32 |  |  |  |  | .63 |
|  | PS33 |  |  |  |  | .67 |
|  | PS34 |  |  |  |  | .63 |
| PS4 | PS41 |  |  |  |  | .27 |
|  | PS42 |  |  |  |  | .73 |
|  | PS43 |  |  |  |  | .64 |
|  | PS44 |  |  |  |  | .65 |
| PS5 | PS51 |  |  |  |  | .28 |
|  | PS52 |  |  |  |  | .73 |
|  | PS53 |  |  |  |  | .77 |
|  | PS54 |  |  |  |  | .67 |
| PS6 | PS61 |  |  |  |  | .71 |
|  | PS62 |  |  |  |  | .66 |
|  | PS63 |  |  |  |  | .75 |
|  | PS64 |  |  |  |  | .54 |
| PS7 | PS71 |  |  |  |  | .70 |
|  | PS72 |  |  |  |  | .36 |
|  | PS73 |  |  |  |  | .58 |
|  | PS74 |  |  |  |  | .65 |
| *Note.* PPSU = parental problematic smartphone use, DCT = difficult child temperament, PS = parenting stress. PS1 to PS7 are the seven latent dimensions of the construct parenting stress. | | | | | | |

**Table A.2**Moderated mediation model.

|  | ***β*** | **SE** | **95% CI** | ***p*** | ***R^2^*** |
| --- | --- | --- | --- | --- | --- |
| **Model examining factors associated with parenting stress** | | | | | .18 |
| DCT (a-path association) | .42 | .11 | [.25, .68] | .000 |  |
| **Model examining factors associated with parental problematic smartphone use** | | | | | .19 |
| DCT (c’-path; direct association) | .01 | .11 | [-.19, .20] | .956 |  |
| PS (b-path association) | .42 | .09 | [.25, .60] | .000 |  |
| gend | .07 | .14 | [-.12, .45] | .252 |  |
| PS×gend (moderation effect) | -.03 | .07 | [-.18, .10] | .607 |  |
| **Indirect association**  **Total association** | .18  .18 | .06  .09 | [.08, .32]  [.03, .38] | .001  .023 |  |
| *Note.* PPSU = parental problematic smartphone use, DCT = difficult child temperament, PS = parenting stress, gend = gender. | | | | | |
